# Autophagy promotes degradation of internalized collagen and regulates distribution of focal adhesions to suppress cell adhesion

**DOI:** 10.1242/bio.027458

**Published:** 2017-10-02

**Authors:** Shinichi Kawano, Takehiro Torisu, Motohiro Esaki, Kumiko Torisu, Yuichi Matsuno, Takanari Kitazono

**Affiliations:** Department of Medicine and Clinical Science, Graduate School of Medical Sciences, Kyushu University, Fukuoka 812-8582, Japan

**Keywords:** Atg5, Atg7, Extracellular matrix

## Abstract

Adhesion of cells to the extracellular matrix (ECM) via focal adhesions (FAs) is crucial for cell survival, migration, and differentiation. Although the regulation of FAs, including by integrins and the ECM, is important to cell behavior, how FAs are regulated is not well known. Autophagy is induced by both cell adhesion and cell detachment. Here, we showed that autophagosomes are located close to internalized collagen and paxillin, which is a well-known marker of FAs. Autophagy-deficient cells showed increased levels of internalized collagen compared with control cells. Moreover, paxillin exhibited a more peripheral distribution and the area of paxillin was increased, and adhesion-induced focal adhesion kinase signaling was impaired and adhesion was enhanced, in autophagy-deficient cells. These results suggest that autophagy suppressed cell adhesion by regulating internalized ECM and FAs.

## Abstract (precis)

**Summary:** Autophagosomes localized close to internalized collagen and focal adhesions. In autophagy-deficient cells, internalized collagen and focal adhesion were increased, focal adhesion kinase signaling was impaired, and cell adhesion was enhanced.

## INTRODUCTION

Adhesion of cells to the extracellular matrix (ECM) plays an important role in the regulation of cellular morphology, migration, proliferation, survival, and differentiation (Huveneers and Danen, 2009). Cell–matrix adhesion is crucial during development, and for tissue maintenance and induction of tissue repair. Focal adhesions (FAs) are sites where a cell engages with the ECM; they not only function as scaffolds but also contribute to signaling that regulates cell structure, dynamics, and fate. FAs are enriched in integrins and in cytoskeletal and signal proteins such as paxillin, vinculin, and focal adhesion kinase (FAK) (Winograd-Katz et al., 2014). Integrins are the principle cell-surface receptors involved in cell adhesion for integration with the ECM. Integrins are activated by binding of the ECM to cells at FAs in the process of cell–ECM adhesion (Berrier and Yamada, 2007). Active integrins and integrin ligands undergo endocytic tracking and induce FAK autophosphorylation at tyrosine 397, which creates a binding site for the Src-homology (SH) 2 domain of Src (Mitra et al., 2005). Phosphorylation of FAK at tyrosine 397 plays an important role in FA disassembly and is an established marker of activated FAK at FAs (Ezratty et al., 2005). The activated FAK–Src complex stimulates downstream signaling and regulates the activity of several members of the Rho family of small GTPases. As a result, stimulation of FAs by the ECM enhances cell adhesion and results in cell spreading (Nagano et al., 2012).

Macroautophagy, hereafter termed autophagy, plays an indispensable role in the intracellular degradation of proteins and organelles. Although starvation is the most extensively studied condition that induces autophagy, the process can be induced in response to several physiological and pathological conditions (Torisu et al., 2013). The ECM can modulate autophagic signaling pathways, as several ECM constituents induce autophagy (Neill et al., 2014). In a study of cell migration, autophagy was reduced at the leading edge of cells compared with the rear edge (Tuloup-Minguez et al., 2013). Mice lacking the collagen VI gene exhibited muscular dystrophies caused by defective autophagy (Grumati et al., 2010). Cell detachment from the matrix has been demonstrated as a trigger of autophagy, and autophagy protected cells from anoikis (Avivar-Valderas et al., 2011; Fung et al., 2008). Conversely, some reports suggest that attachment induces autophagy. Blocking integrin with antibodies reduced the extent of starvation-induced autophagy (Edick et al., 2007). The integrin ligand has been shown to stimulate autophagy through integrins in a process mediated by p38-MAPK (Zheng et al., 2012); however the role of attachment-induced autophagy is not well understood.

Internalized integrins and integrin ligands are trafficked to the endosome. Some of the integrins are degraded, but the majority of internalized integrins are recycled back to the plasma membrane (Bridgewater et al., 2012). Although collagen has been shown to be localized in lysosomes in cells treated with a lysosome inhibitor (Everts et al., 1996), it is not well understood whether internalized integrin ligands are also degraded via autophagy.

To address this issue, we demonstrate here that autophagosomes are located close to the internalized ECM and internalized complexes of FAs in the cell. Collagens are the main constituent of ECM. *Atg5* and *Atg7* are essential genes for autophagosome formation (Kuma et al., 2004; Komatsu et al., 2005). Here, we show using *Atg5*- and *Atg7*-deficient cells that autophagy regulates the distribution of FAs and FA signaling, and thus regulates cell adhesion and cell spreading.

## RESULTS

### Autophagosomes colocalized with internalized ECM

**Fig. 1.**
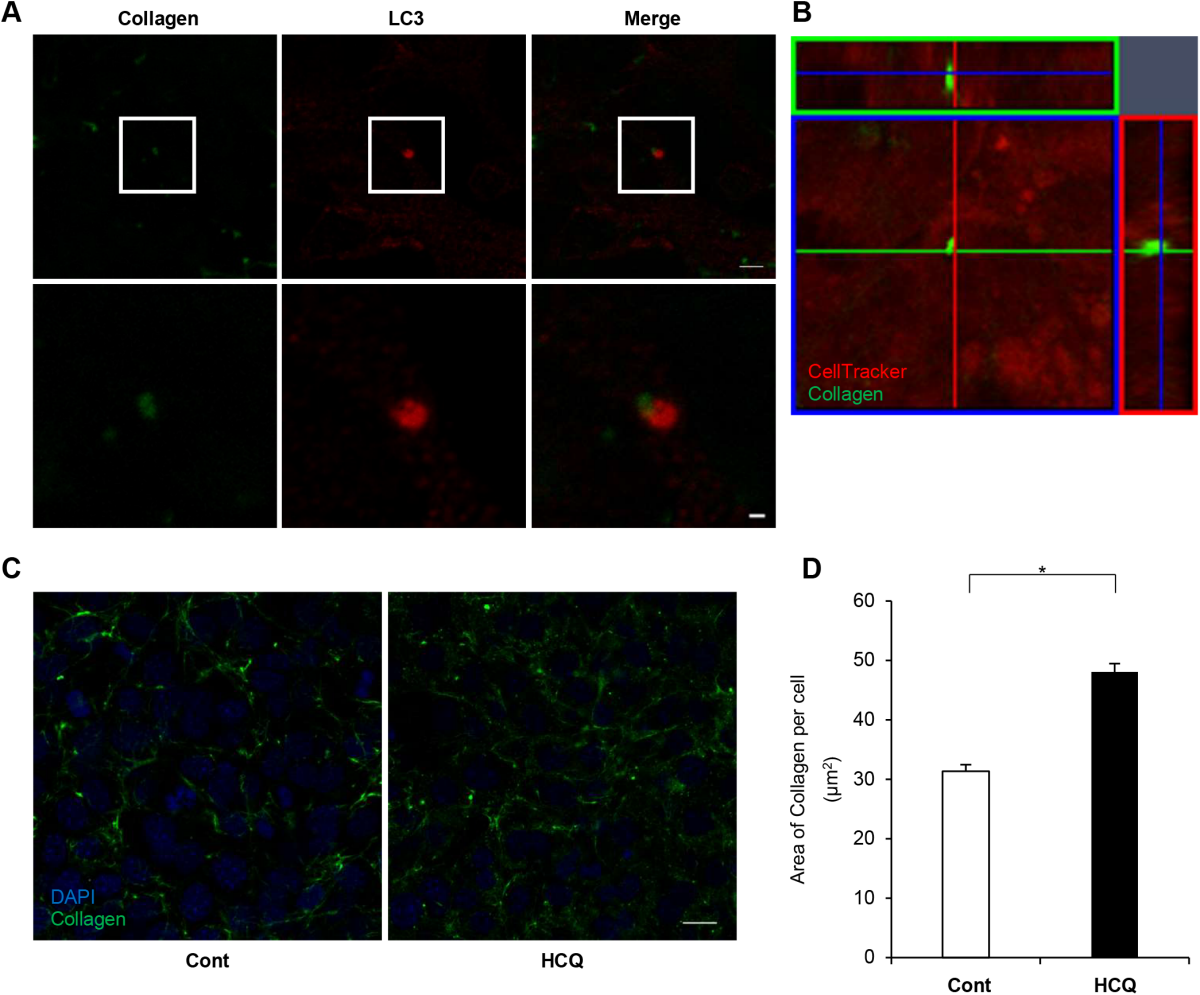
**Autophagosomes associated with collagen.** (A) Representative confocal images of collagen (left), LC3 (middle), and merged images (right). Lower panels show enlarged images of the boxed regions in the upper panels. The fibroblasts in these images were cultured on FITC-labeled collagen and stained with anti-LC3 antibody. Scale bars: 5 µm (upper) and 1 µm (lower). (B) Representative image of orthographic project. Fibroblasts were cultured on FITC-labeled collagen and stained with a CellTracker orange probe. (C) Collagen deposition with (right) and without (left) HCQ treatment. Fibroblasts were cultured on FITC-labeled collagen for 120 min. Scale bar: 20 µm. (D) Quantification of the area of FITC-labeled collagen per cell. Data are mean and s.e.m. in control and HCQ-treated cells (*n*=3 fields of control; five fields for HCQ-treated cells). *P<0.001, Student's *t*-test. Three independent similar experiments are shown.

We cultured fibroblasts for 24 h on culture dishes coated with fluorescein isothiocyanate (FITC)-labeled collagen. The fibroblasts took up the FITC-labeled collagen. The internalized collagen localized close to microtubule-associated protein 1-light chain 3 (LC3), an established autophagosome marker (Fig. 1A). Analysis of 13 random fields revealed that FITC-labeled collagen localized close to LC3 in 14% (24/171) of cells. To examine the distribution of collagen, cells were cultured on FITC-collagen and stained with a CellTracker probe. FITC-collagen was observed as dots in an intracellular distribution (Fig. 1B). To examine involvement of the autophagy–lysosome pathway in the degradation of internalized collagen, we treated the fibroblasts with hydroxychloroquine (HCQ), an inhibitor of autophagic flux (Fig. 1C). Collagen deposition was increased after HCQ treatment (Fig. 1D).

### Internalized collagen accumulated in autophagy-deficient fibroblasts

**Fig. 2.**
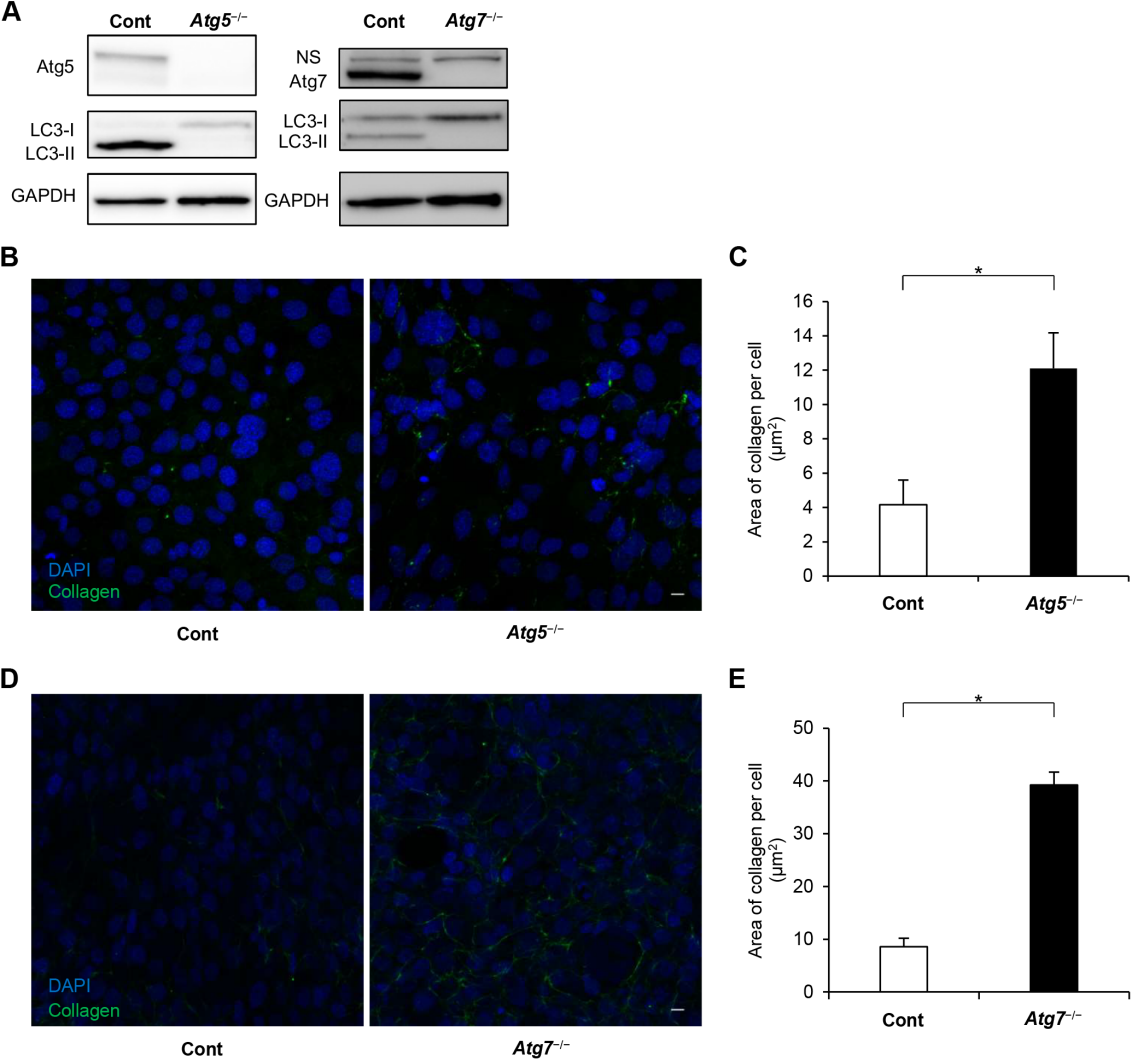
**Internalized collagen levels were higher in autophagy-deficient cells than in control cells.** (A) Left panels show western blot analysis of Atg5 (Atg5–Atg12 conjugate), LC3-I and LC3-II in control and *Atg5*^−/−^ MEFs. Right panels show western blot analysis of Atg7, LC3-I, and LC3-II in control and *Atg7*^−/−^ MEFs. NS, non-specific. GAPDH was used as an internal control. (B) Internalized FITC-labeled collagen in control (left) and *Atg*5^−/−^ (right) MEFs. The cells were cultured on FITC-labeled collagen for 120 min. Scale bar: 20 µm. (C) Quantification of the FITC-labeled collagen area per cell. Data presented are mean and s.e.m. in control and *Atg5*^−/−^ cells. *P<0.05, Student's *t*-test (*n*=5 fields of control; seven fields for *Atg5*^−/−^ cells). Four independent similar experiments are shown. (D) Internalized FITC-labeled collagen in control (left) and *Atg*7^−/−^ (right) MEFs. The cells were cultured on FITC-labeled collagen for 120 min. Scale bar: 20 µm. (E) Quantification of the FITC-labeled collagen area per cell. Data presented are mean and s.e.m. in control and *Atg7*^−/−^ cells. *P<0.05, Student's *t*-test (*n*=10).

**Fig. 3.**
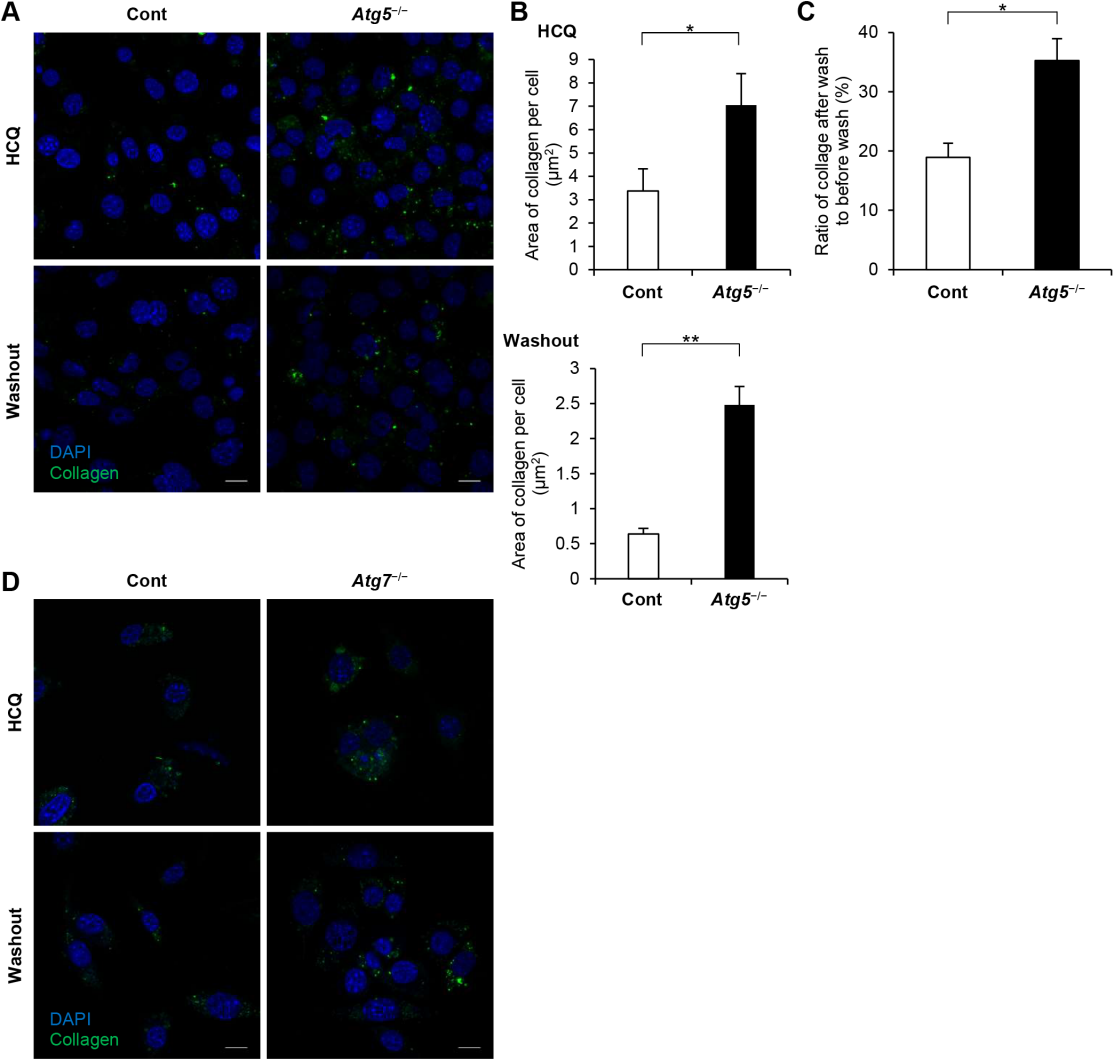
**Degradation of internalized collagen was decreased in autophagy-deficient cells compared with control cells.** (A) Representative confocal images of internalized collagen in control and *Atg5*^−/−^ MEFs. These MEFs were pre-cultured on FITC-labeled collagen in the presence of HCQ (40 µM) for 16 h, then were transferred to new chamber slides without FITC-labeled collagen. Subsequently, the cells were cultured for 24 h with (upper panel) or without (lower panel) HCQ. Scale bar: 20 µm. (B) Quantification of the area of FITC-labeled collagen per cell in cells treated with HCQ (upper graph) and after washout of HCQ (lower graph). The bar graph for HCQ shows mean±s.e.m. in control and *Atg5*^−/−^ cells and the graph for washout shows mean±s.e.m. in control and *Atg5*^−/−^ cells (*n*=7 fields of control; six fields for *Atg5*^−/−^ cells). Three independent similar experiments are shown. *P<0.05, ** *P*<0.001, Student's *t*-test. (C) Internalized collagen remaining after washout of HCQ, as a percentage of internalized collagen in HCQ-treated cells. *P<0.01, Student's *t*-test; mean±s.e.m. (D) Representative confocal images of internalized collagen in control and *Atg7*^−/−^ MEFs. After pre-culture on FITC-labeled collagen in the presence of HCQ (40 µM) for 16 h, cells were cultured for 24 h with (upper panel) or without (lower panel) HCQ on new chamber slides without FITC collagen. Scale bar: 20 µm.

Atg5 and Atg7 are essential molecules for the induction of autophagy (Kuma et al., 2004; Komatsu et al., 2005). We therefore used *Atg5*- and *Atg7*-deficient murine embryonic fibroblasts (MEFs) to examine whether autophagy deficiency affected the amount of internalized collagen. We initially confirmed the absence of Atg5 (Atg5–Atg12 conjugate) in *Atg5*^−/−^ cells and the absence of Atg7 in *Atg7*^−/−^ cells by western blotting (Fig. 2A). In both *Atg5*^−/−^ cells and *Atg7*^−/−^ cells, the ratio of LC3-I to LC3-II was consistent with impaired autophagy (Fig. 2A). In *Atg5*^−/−^ cells, the amount of internalized FITC-labeled collagen was greater than that in control cells (Fig. 2B,C). This increased internalized collagen in autophagy-deficient cells was also reproduced using *Atg7*^−/−^ cells (Fig. 2D,E). To test whether this increased collagen level was due to increased uptake or decreased degradation in *Atg5*^−/−^ cells, fibroblasts were pre-cultured with HCQ on a FITC-labeled collagen-coated dish for 24 h for collagen uptake, and were then transferred to a new dish without FITC-labeled collagen to avoid further uptake (Fig. 3A). The amount of internalized FITC-labeled collagen was significantly greater in autophagy-deficient cells than in control cells, both with and without HCQ treatment (Fig. 3B). Moreover, the percentage of the internalized FITC-labeled collagen that remained after washout of HCQ was higher in *Atg5*^−/−^ cells than in control cells (Fig. 3C). We reproduced similar findings using *Atg7*^−/−^ cells, in which the amount of collagen was greater in *Atg7*^−/−^ cells compared with control cells, especially without HCQ treatment (Fig. 3D). These results suggest that the difference in the amount of FITC-labeled collagen between control and autophagy-deficient cells was more dependent on impaired degradation than on elevated uptake in autophagy-deficient cells.

### Autophagy regulated focal adhesion and subsequent signaling

**Fig. 4.**
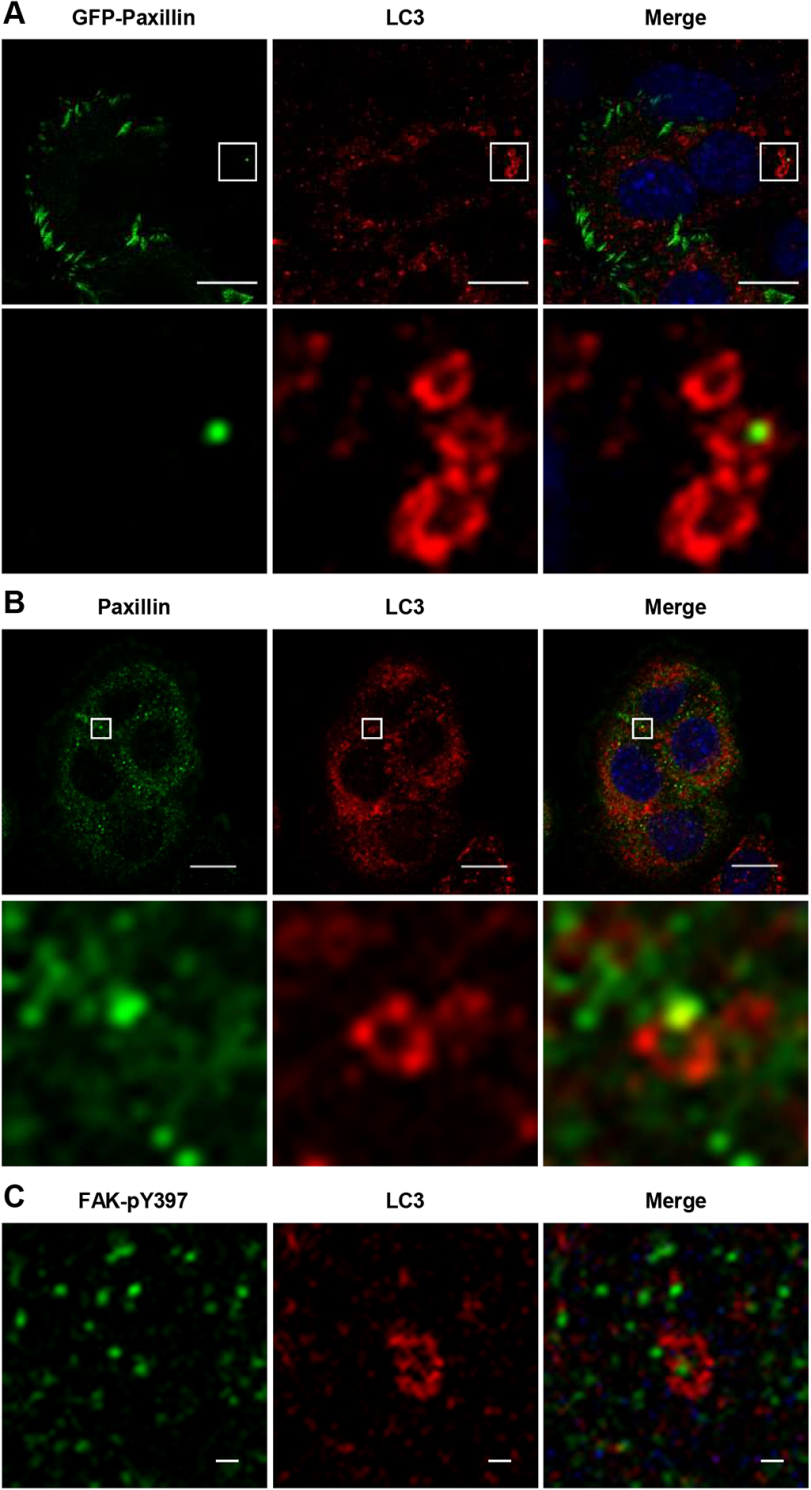
**Autophagosomes associated with the internalized complexes of focal adhesion (FA) complex.** (A) Representative confocal image of GFP-paxillin (left) and endogenous LC3 (middle). Lower panels show enlarged images of the boxed regions in the upper images. Paxillin was surrounded by puncta of LC3. Whole-cell merged image with DAPI counterstaining (right). Scale bar: 10 µm. (B) Fibroblasts were plated, and after 90 min were stained with antibodies against paxillin (left), LC3 (middle) and whole-cell merged image with DAPI counterstaining (right). The lower panels show enlarged images of the boxed regions in the upper images. Scale bar: 10 µm. (C) Representative confocal image of FAK-pY397 (left), LC3 (middle), and whole-cell merged image with DAPI counterstaining (right). Scale bar: 1 µm.

**Fig. 5.**
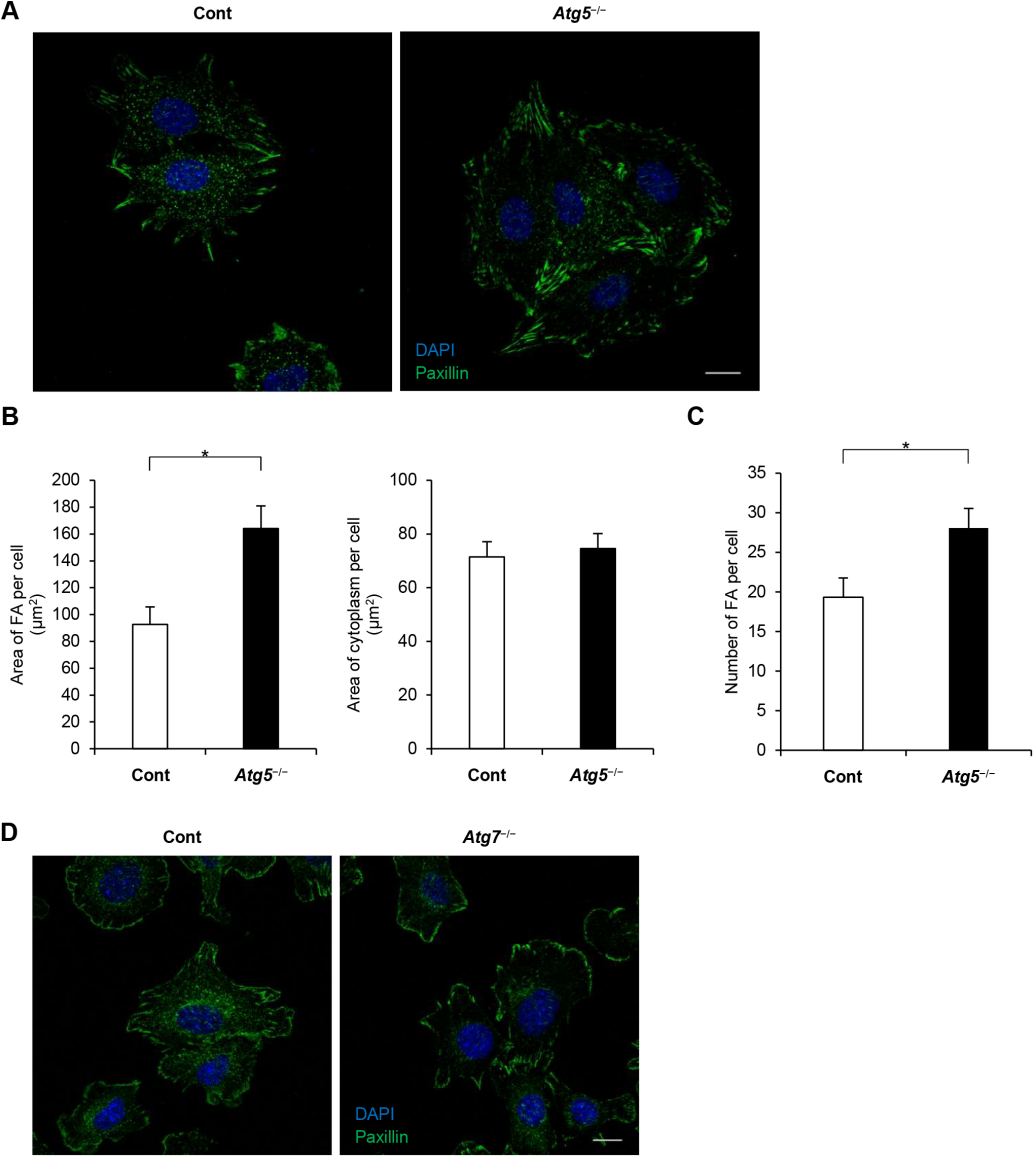
**FA complex distribution in autophagy-deficient cells and control cells.** (A) Control and *Atg5*^−/−^ MEFs were cultured for 120 min and stained with anti-paxillin antibody and with DAPI. Scale bar: 20 µm. (B) Area of paxillin, presented as mean and s.e.m, in control and *Atg5*^−/−^ cells (*n*=8 fields of control; nine fields for *Atg5*^−/−^ cells). *P<0.01, Student's *t*-test. Four independent similar experiments are shown. (C) The number of FAs in control and *Atg5*^−/−^ cells, presented as mean and s.e.m, in control and *Atg5*^−/−^ cells. *P<0.01, Student's *t*-test. (D) Representative confocal image of control and *Atg7*^−/−^ MEFs, which were cultured for 120 min and stained with anti-paxillin antibody and DAPI. Scale bar: 20 µm.

The ECM controls cell adhesion through FAs (Turner, 2000). We analyzed the colocalization of FAs and autophagosomes. The expression of the paxillin-EGFP fusion protein was engulfed in LC3 (Fig. 4A). This result was confirmed by immunocytochemical staining of endogenous paxillin and LC3. This revealed that paxillin was surrounded by puncta of LC3 (Fig. 4B). Similarly, immunocytochemistry showed that FAK-pY397 was surrounded by LC3 (Fig. 4C). To address whether this colocalization had functional relevance, we analyzed paxillin localization in autophagy-deficient cells. In control cells, paxillin was distributed in punctate form in the cytoplasm and at the cell periphery, whereas in *Atg5*^−/−^ cells it was distributed more at the cell periphery (Fig. 5A). The total area of FAs per cell was larger in *Atg5*^−/−^ cells than in control cells, although the non-FA area per cell was comparable in control and *Atg5*^−/−^ cells (Fig. 5B). The number of FAs was significantly larger in *Atg5*^−/−^ cells than in control cells (Fig. 5C). Similarly, in *Atg7*^−/−^ cells, paxillin was distributed more at the cell periphery than in control cells (Fig. 5D). These results indicate that more FAs are retained in autophagy-deficient cells.

**Fig. 6.**
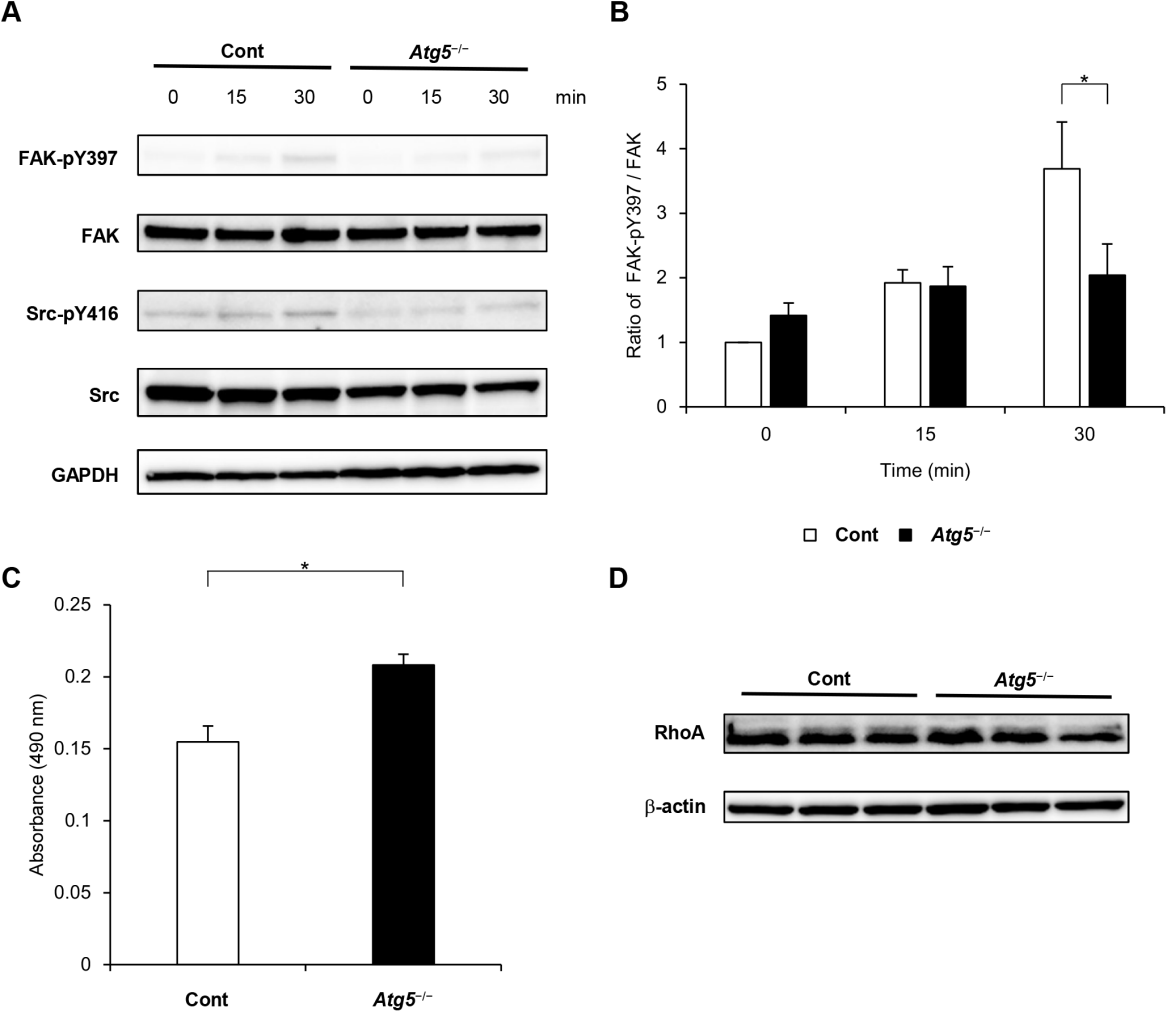
**Inhibiting autophagy attenuated FA kinase (FAK) and enhanced RhoA activity.** (A) Control and *Atg5*^−/−^ MEFs were plated on collagen I-coated dishes for 0, 15 and 30 min. Cell lysates were collected from dishes at the indicated time points. Lysates were immunoblotted with anti-FAK-pY397, anti-FAK, anti-Src-pY416, and anti-Src antibodies. GAPDH is shown as a loading control. (B) Quantification of the ratio of FAK-pY397 and FAK protein levels. Bar graph shows mean and s.e.m. (*n*=10 replicates). *P<0.05, ANOVA with Tukey's post hoc test. (C) RhoA activity in control and *Atg5*^−/−^ MEFs. Control and *Atg5*^−/−^ MEFs were plated on collagen I for 30 min. Cell lysates were assessed by G-LISA RhoA activation assay. The graph shows absorbance at 490 nm from five experiments (*n*=5 replicates). *P<0.01, Student's *t*-test. Three independent similar experiments are shown. (D) Western blot analysis of RhoA in control and *Atg5*^−/−^ MEFs. Actin was used as a loading control.

Next, we investigated FA signaling. FAK and Src protein levels did not differ markedly between control and *Atg5*^−/−^ cells, even after adhesion. FAK phosphorylation at tyrosine 397 is a marker of activated FAK in cell adhesion (Ezratty et al., 2005), and phosphorylation of Src at tyrosine 416 in the activation loop of the kinase domain upregulates its enzyme activity (Mitra et al., 2005). FAK phosphorylation at tyrosine 397 and Src phosphorylation at tyrosine 416 were increased after cell adhesion to collagen in control cells (Fig. 6A). Although Src phosphorylation was comparable between control and *Atg5*^−/−^ cells, FAK phosphorylation was significantly attenuated in *Atg5*^−/−^ cells at 30 min after cell adhesion (Fig. 6B).

FAK and RhoA regulate each other (Ren et al., 2000), and we therefore analyzed RhoA activity. RhoA activity was higher in *Atg5*^−/−^ cells than in control cells plated on collagen-coated dishes for 30 min (Fig. 6C), although total RhoA protein levels did not differ between *Atg5*^−/−^ and control cells (Fig. 6D).

### Autophagy suppressed cell attachment

**Fig. 7.**
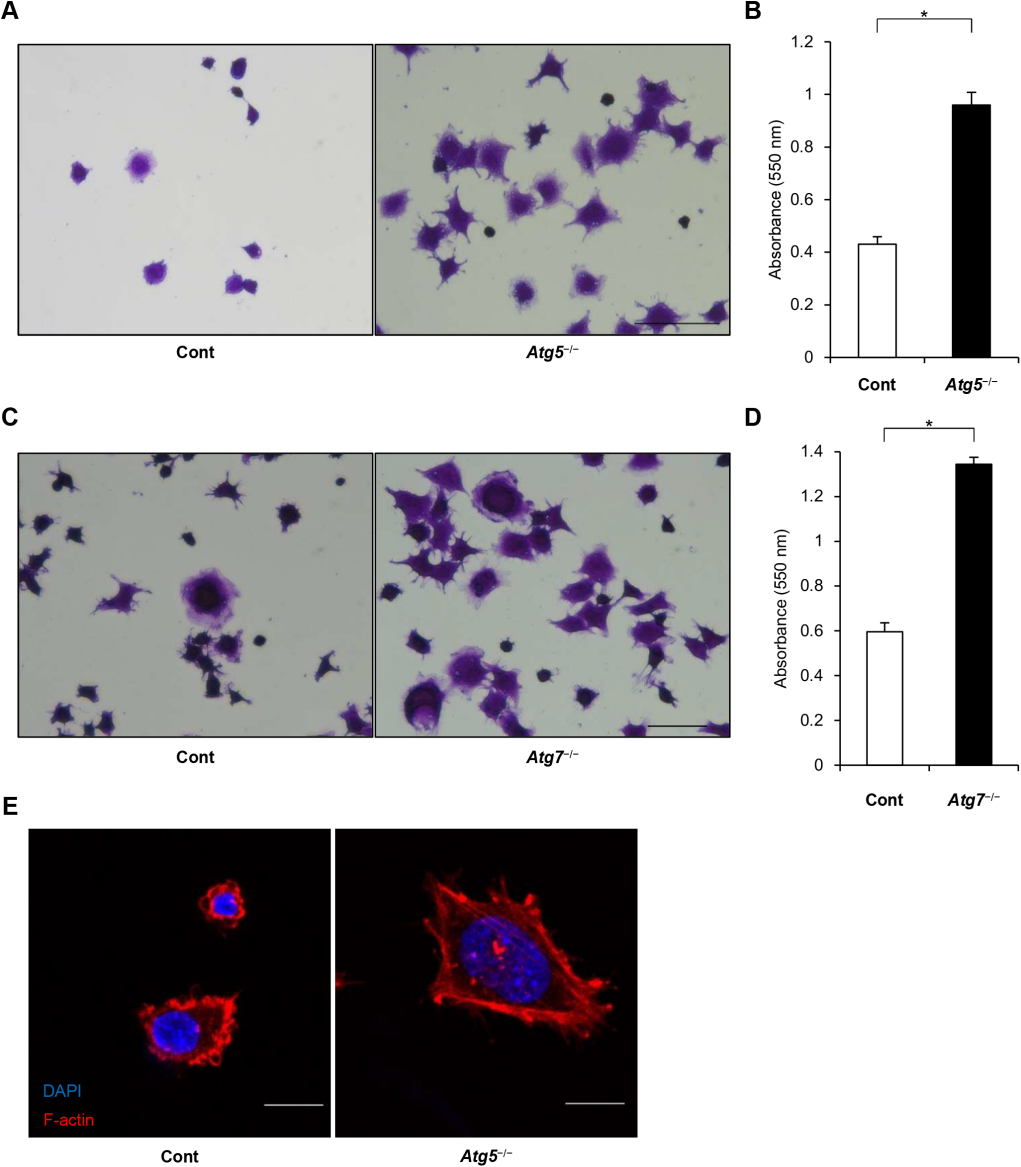
**Autophagy-deficient cells exhibited more adherence than control cells.** (A) Control and *Atg*5^−/−^ MEFs were cultured on collagen for 30 min and stained with 0.5% crystal violet. Scale bar: 50 µm. (B) Adhesion assay in control and *Atg5*^−/−^ MEFs. Crystal violet staining in these cells was eluted and absorbance of the resulting solution at 550 nm was examined. Data presented are from three experiments. *P<0.01, Student's *t*-test. (C) Representative crystal violet staining images of control and *Atg*7^−/−^ MEFs. Scale bar: 50 µm. (D) Adhesion assay in control and *Atg7*^−/−^ MEFs. Data presented are from three experiments. *P<0.01, Student's *t*-test. (E) Representative images of phalloidin staining for F-actin and DAPI staining of control and *Atg*5^−/−^ MEFs. The cells were cultured on collagen for 120 min. Scale bar: 10 µm.

Finally, to address whether the molecular differences observed between control and *Atg5*^−/−^ cells influenced cell behavior, we conducted an adhesion assay. Thirty minutes after plating on collagen-coated dishes, more autophagy-deficient cells than control cells had adhered to the collagen (Fig. 7A). This result was consistent with a previous study using atg7 or atg12 knockdown cells (Kenific et al., 2016). When we examined cell morphology, we observed more cell spreading among the *Atg5*^−/−^ cells than the control cells (Fig. 7B). We reproduced similar findings using *Atg7*^−/−^ cells. We observed more cell spreading among the *Atg7*^−/−^ cells than the control cells (Fig. 7C,D). Consistent with this finding, F-actin filaments in the *Atg5*^−/−^ cells were longer than those in the control cells (Fig. 7E).

## DISCUSSION

Our data suggest that autophagy is involved in the degradation of internalized collagen and the distribution of FAs to suppress cell adhesion in fibroblast cells. Degradation of collagen in fibroblasts is a fundamental process in tissues under both physiological and pathological conditions (Everts et al., 1996). There are two pathways for collagen degradation: an extracellular pathway and an intracellular pathway. The extracellular pathway involves cleavage of collagen fibrils by matrix metalloprotease enzymes (McKleroy et al., 2013). In the intracellular pathway in fibroblasts, the engulfment of collagen fibrils is mediated by integrins (Dupuy and Caron, 2008). Collagen internalized from the ECM by fibril phagocytosis is degraded in the lysosomal network (Everts et al., 1996). Our data demonstrate that autophagosomes were located close to intracellular collagen, and that inhibiting autophagy increased levels of internalized collagen. These results suggest that autophagy is involved in the process of internalizing collagen degradation in the lysosome.

We showed that autophagy regulated FAK phosphorylation upon cell adhesion to collagen. It has been reported that autophagy suppresses FAK signaling under specific conditions, such as when FAK is deleted (Sandilands et al., 2011). Recently, it was demonstrated that FAK activation and subsequent downstream signaling were dependent on the endocytosis of active integrins and integrin ligands (Alanko and Ivaska, 2016; Alanko et al., 2015). Autophagy has been shown to maintain the endosome membrane (Kreibich et al., 2015), and an overlap between the autophagic and endocytotic pathways was demonstrated (Tooze et al., 2014). In autophagy-deficient cells, endosomal dysfunction may be associated with attenuated FAK activation. Furthermore, FAK and RhoA regulate each other. FAK has been shown to suppress RhoA activity (Ren et al., 2000), and RhoA was found to induce FAK activation (Del Re et al., 2008). Impaired degradation of active RhoA has been reported in lysosomal v-ATPase-deficient cells in which autophagosome degradation was inhibited, accompanied by enhanced RhoA activity (Belaid et al., 2013). Consistent with previous reports, our data show that although total RhoA protein levels were comparable in *Atg5*^−/−^ and control cells, RhoA activity was elevated in *Atg5*^−/−^ cells compared with control cells. Our results suggest that impaired FAK signaling in autophagy-deficient cells resulted in enhanced RhoA activation. In FAK-deficient cells, FA turnover has been shown to be suppressed (Ren et al., 2000; Webb et al., 2004). In RhoA-overexpressing cells, the FA marker surface-associated vinculin was enriched (Cáceres et al., 2005). We demonstrated that paxillin and active form of FAK was engulfed in autophagosomes, and that the morphology of engulfed paxillin was mostly of a round form. We demonstrated that endogenous paxillin exhibited more of a cell surface-associated distribution pattern in autophagy-deficient cells compared with control cells. We also observed that an autophagy deficiency promoted adhesion and resulted in more cell spreading during the adhesion period. These results suggest that autophagy regulates cell motility via FA signaling. One elegant study showed that FA lifetime was increased in *Atg7* and *Atg12* knockdown cells compared with control cells using fluorescence-labeled paxillin overexpression (Kenific et al., 2016). Another study showed that autophagy interacted with and then degraded paxillin to promote FA disassembly (Sharifi et al., 2016). These reports are consistent with our results.

In metastasis, cell–matrix adhesion is key to allowing cells to escape from their primary sites, and is required for them to be able to colonize secondary sites. Many studies have shown an association between autophagy and cancer metastasis (Mowers et al., 2017). Autophagy requires several processes and features involved in metastasis, including stem-like phenotype (Mowers et al., 2017) and protection from anoikis (Fung et al., 2008). Cell–matrix adhesion regulated by autophagy, as demonstrated in this report, may be one of the mechanisms underlying the relationship between autophagy and metastasis.

In summary, autophagosomes are located close to internalized collagen and internalized complexes of FAs. Autophagy enhances FAK signaling and regulates FAs to suppress cell adhesion.

## MATERIALS AND METHODS

### Cell culture

Control, *Atg5*^−/−^, and *Atg7*^−/−^ MEFs purchased from Riken Cell Bank (Tsukuba, Japan) were cultured in Dulbecco's modified Eagle's medium (DMEM) containing 10% fetal bovine serum (FBS, Sigma-Aldrich) and 1% penicillin and streptomycin (GIBCO) at 37°C with 5% CO_2_ (Kuma et al., 2004). For the adhesion assay (Hu et al., 2008), the Rho activation assay (Ren et al., 2000) and western blotting (Cheng et al., 2014), cells were grown to 60% confluence and trypsinized with 0.05% tripsin-EDTA (Gibco, Thermo Fisher Scientific, MA, USA). The trypsinization was stopped by addition of 0.5 mg/ml soybean trypsin inhibitor (Wako, Osaka, Japan) in DMEM. The cells were pelleted and washed once more with 0.5 mg/ml soybean trypsin inhibitor, followed by another wash with serum-free medium, then were suspended in DMEM containing 0.1% bovine serum albumin (BSA, Sigma) and maintained in suspension for 1 h at 37°C (Cheng et al., 2014). The suspended cells (2×10^5^ cells/ml) were plated on collagen I–coated dishes (Corning, NY, USA) and incubated at 37°C. EGFP-m-Paxillin (Plasmid #80023) was obtained from Addgene. We transfected 1 µg plasmid into 2×10^4^ MEFs using Lipofectamine® 2000 reagent (Thermo Fisher Scientific) according to the manufacturer's protocol.

### Antibodies

The following antibodies were used for immunoblotting: anti-LC3 (L8918, Sigma-Aldrich), anti-paxillin (ab32084, Abcam), anti-Atg5 (#12994, Cell Signaling), anti-Atg7 (A2856, Sigma-Aldrich), anti-glyceraldehyde 3-phosphate dehydrogenase (GAPDH; ab181602, Abcam), anti-FAK-pY397 (44-624G, ThermoFisher Scientific), anti-FAK (610087, BD Transduction Laboratories), anti-Src-pY416 (#2101, Cell Signaling), anti-Src (#2109, Cell Signaling), anti-RhoA (ab187027, Abcam), and anti-β-actin (ab8227, Abcam).

### Western blotting

Adherent cells cultured for 24 to 48 h were harvested on ice with a cell scraper. An additional culture of serum-starved cells was trypsinized and kept in suspension for 1 h, then collected in tubes. The cells were the incubated at 37°C for 15 and 30 min, and harvested (Cheng et al., 2014). The cell suspensions were centrifuged at 300 ×***g*** at 4°C for 5 min and the supernatant was discarded. The cell pellets were washed with chilled phosphate-buffered saline (PBS) and lysed in radioimmunoprecipitation assay lysis buffer (Nacalai Tesque, Kyoto, Japan) or NP40 lysis buffer (Wako, Osaka, Japan) containing protease inhibitor (Nacalai Tesque) and phosphatase inhibitor (Nacalai Tesque) cocktails for 5 min on ice. The cell lysate was centrifuged (16000 ×***g*** at 4°C for 15 min). The lysate [10–20 µg protein, as measured with a BCA Protein Assay kit (Thermo Fisher Scientific)] was then mixed with SDS sample buffer (Nacalai Tesque), separated by SDS-PAGE using pre-made 7.5% or 5–20% polyacrylamide gel plates (e-PAGEL, Atto, Tokyo, Japan), transferred to iBlot® 2 Transfer Stacks PVDF mini membranes using an iBlot® 2 Dry Blotting system (Thermo Fisher Scientific), and immunoblotted with specific antibodies at 1:1000 to 1:5000 dilution (Alanko et al., 2015; Torisu et al., 2013).

### Immunofluorescence microscopy

For immunofluorescence microscopy, cells were grown on 4-well chamber slides (Lab-Tek, Thermo Fisher Scientific) that were pre-coated with 1 µg/cm^2^ of fibronectin (Sigma-Aldrich, Germany), 1 µg/cm^2^ of collagen (Sigma), or 1 µg/cm^2^ of FITC-conjugated collagen I (4001, Chondrex, WA, USA) per well (Torisu et al., 2013, 2016). To label whole cells, they were incubated with 1 µM of CellTracker (Thermo Fischer Science) orange fluorescent probe according to the manufacturer's protocol. The cells were then fixed with 4% paraformaldehyde in PBS (pH 7.4) for 10 min at room temperature, and permeabilized for 5 min with PBS containing 0.1% Triton X-100. Cells were incubated with Blocking One (Nacalai Tesque) for 30 min and incubated with specific antibodies at 1:50 to 1:250 dilution overnight at 4°C. We visualized F-actin polymerization via phalloidin staining (A34055, Thermo Fisher Scientific). The cells were then incubated with secondary antibody for 30 min, and mounted in VECTASHIELD Mounting Medium with DAPI (Vector Laboratories, Burlingame, CA, USA). Immunofluorescence samples were examined by confocal microscopy using a Zeiss LSM 700 microscope (Carl Zeiss MicroImaging, Germany) (Torisu et al., 2013).

### FA size analysis

We used endogenous paxillin as an FA marker (Sandilands et al., 2011; Sharifi et al., 2016). Image analysis was performed using ImageJ software (Wayne Rasband; the Research Services Branch, National Institute of Mental Health, Bethesda, MD, USA) after appropriate thresholding, as previously described (Sharifi et al., 2016).

### Rho-activation assay

Rho-activation was assayed using a RhoA G-LISA kit (Cytoskeleton, Denver, CO, USA). Starved cells were trypsinized and kept in suspension for 1 h then incubated in a dish at 37°C for 30 min, and harvested (Cheng et al., 2014). The RhoA G-LISA assay was performed according to the manufacturer's protocol.

### Adhesion assay

The adhesion assay was performed as previously described (Hu et al., 2008). Briefly, serum-starved cells were trypsinized and kept in suspension for 1 h, then incubated on collagen I-coated dishes at 37°C for 30 min. The cells were fixed with 4% paraformaldehyde, then stained with 0.5% crystal violet in 20% ethanol (Sigma-Aldrich, Germany) for 10 min, washed with ddH_2_O and dried completely. The cells were observed using a Nikon Eclipse Ti-U microscope (Nikon, Tokyo, Japan). Acetic acid (33%) was then added to the dish to dissolve the crystal violet, and absorbance of the resulting solution at 550 nm was examined.

### Statistical analysis

All statistical analyses were performed using JMP Pro 11 (SAS Institute Inc., NC, USA). The statistical significance of differences between groups was evaluated by an unpaired two-tailed Student's *t*-test with Welch's correction, a Mann–Whitney U test, F test repeated-measures analysis of variance (ANOVA), or ANOVA with Tukey's post hoc test. *P*<0.05 was considered statistically significant in all experiments.
